# Bilateral Pneumothorax Complicating Pacemaker Implantation, due to Puncture of the Left Subclavian Vein and Electrode Perforation of the Right Atrium

**DOI:** 10.7759/cureus.11302

**Published:** 2020-11-02

**Authors:** Line Lisbeth Olesen

**Affiliations:** 1 Cardiology, Zealand University Hospital, Roskilde, DNK

**Keywords:** bilateral iatrogenic pneumothorax, complications to pacemaker implantation, prevention of complications to pacemaker implantation, subclavian venous access, risk factors for pneumothorax, atrial lead perforation, electrode perforation and pneumothorax, risk factors for cardiac perforation, case report, myocardial perforation and pneumothorax

## Abstract

Pneumothorax occurs mostly due to needle injury of the pleura when trying to get access to the subclavian vein and rarely due to electrode perforation. The present case report is the first case presented about acute simultaneous iatrogenic bilateral pneumothorax due to puncture of the left subclavian vein and electrode perforation of the atrial wall, the pericardium, and the pleura. Risk factors, and how to avoid these complications, are highlighted, and symptoms, diagnostics, and management of pneumothorax and cardiac perforation are described.

## Introduction

Presentation of a unique case involving iatrogenic bilateral pneumothoraces due to implantation of a pacemaker, on the left side when gaining venous access, on the right side resulting from electrode perforation of the atrial wall, pericardium, pleura, and lung. This is the first case report about the simultaneous occurrence of these complications to pacemaker therapy.

Pneumothorax occurs in 1%-2% of all pacemaker operations, due to needle injury of the pleura typically when attempting to gain access to the subclavian vein [1-5]. Perforation of a pacemaker electrode through the cardiac free wall occurs in 1%-2% of pacemaker implantations, whereas electrode perforation of the pleura resulting in pneumothorax is very rare [5-9] (estimated less than 0.0001% of all pacemaker implantations).

Pacemaker-implanting cardiologists should strive to minimize the incidence of pneumothorax and cardiac perforation in view of the potentially fatal nature of these complications and especially concomitant occurrence, as in the present case.

## Case presentation

A previously healthy 77-year-old female, a nonsmoker, was admitted to the hospital after a month of malaise, tiredness, dizziness, dyspnea, severe headache, and anorexia.

Recently, her general practitioner diagnosed her with arterial hypertension and started administering the diuretic bendroflumethiazide. She received no other medications, no corticosteroids, and no anticoagulants. Her chest X-ray revealed emphysematous lungs without apical bullae.

Upon arrival at the hospital, her blood pressure was 190/50 mmHg, pulse 40-50 bpm, serum-Na 114, and serum-K 3.2 mmol/L. Electrocardiogram (ECG) showed third-degree atrioventricular block and narrow, nodal escape rhythm. CT cerebrum was without any acute changes. Echocardiography was normal. Height was 165 cm, weight 55 kg, and BMI 20 kg/m2.

Bendroflumethiazide was withdrawn, the Na-deficit slowly corrected, and electrolyte derangements were abolished, but the atrioventricular (AV)-block persisted. After implantation of a pacemaker, the blood pressure fell to 160/70 mmHg, she was administered amlodipine, the blood pressure normalized, and the symptoms disappeared. This supported the theory that arterial hypertension with a very high systolic pressure and a wide amplitude was due to initially unnoticed bradycardia and the electrolyte derangements to treatment with the thiazide, conjointly causing the symptoms of the patient.

**Figure 1:**
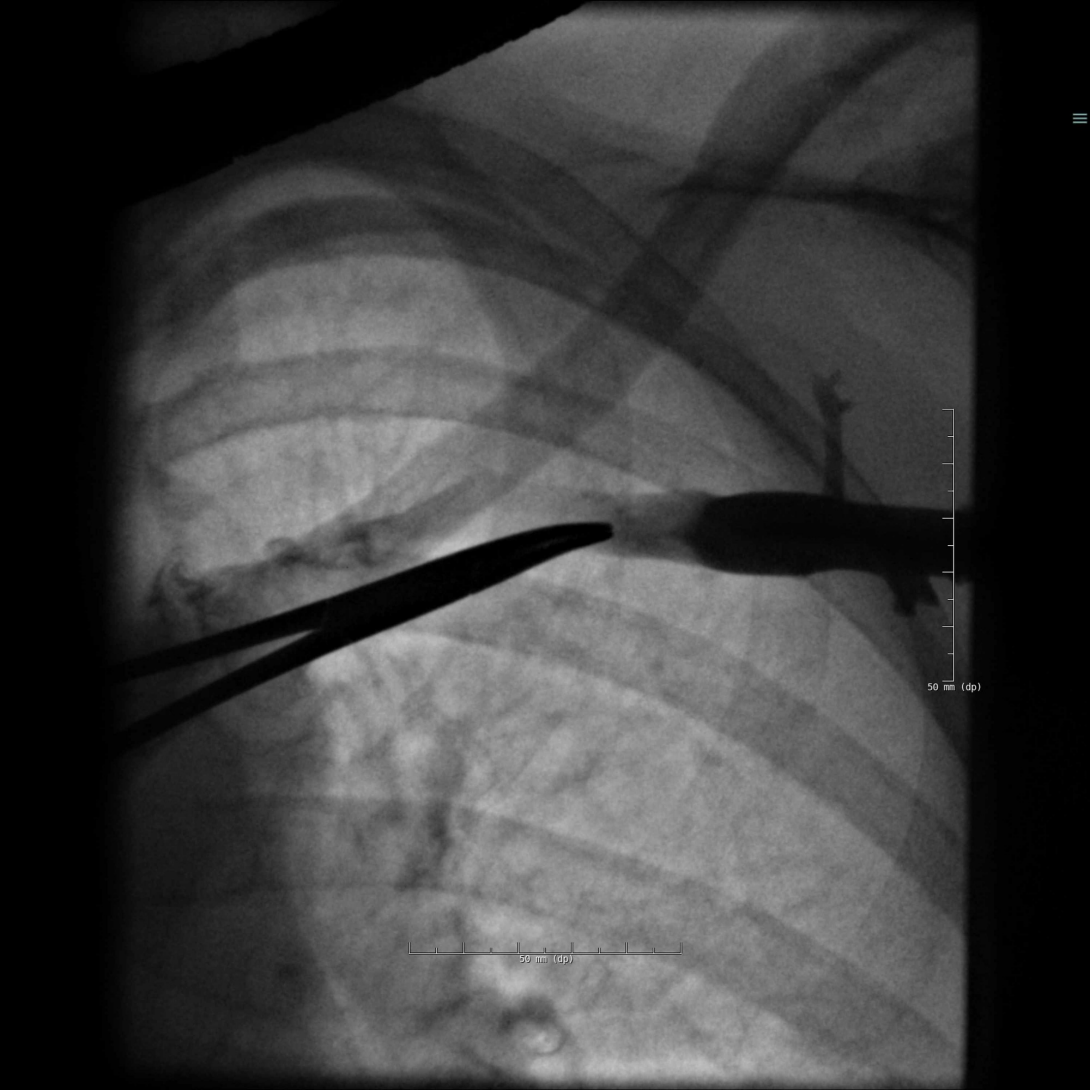
Venography reveals the left subclavian vein.

A dual-chamber (DDD) pacemaker was implanted without immediate complications. Two screw-in electrodes (helical active fixation leads) were inserted via the left subclavian vein. Puncture of the subclavian vein was guided by venography and fluoroscopy, the needle pointing towards the intersection of the clavicle and the first costa (Figure 1).

The vein was punctured in the second attempt, with no aspiration of air, Seldinger technique using two peel-away-sheaths with a diameter of 9 and 7 French to introduce a guidewire into the right atrium and two pacemaker electrodes.

The atrial lead was repositioned a couple of times before good values were obtained on the lateral wall of the right atrium, where it was secured. The ventricular electrode was placed in the apex of the right ventricle. During the implantation, severe problems were not encountered, neither when searching for venous access, nor when fixing the leads.

**Figure 2:**
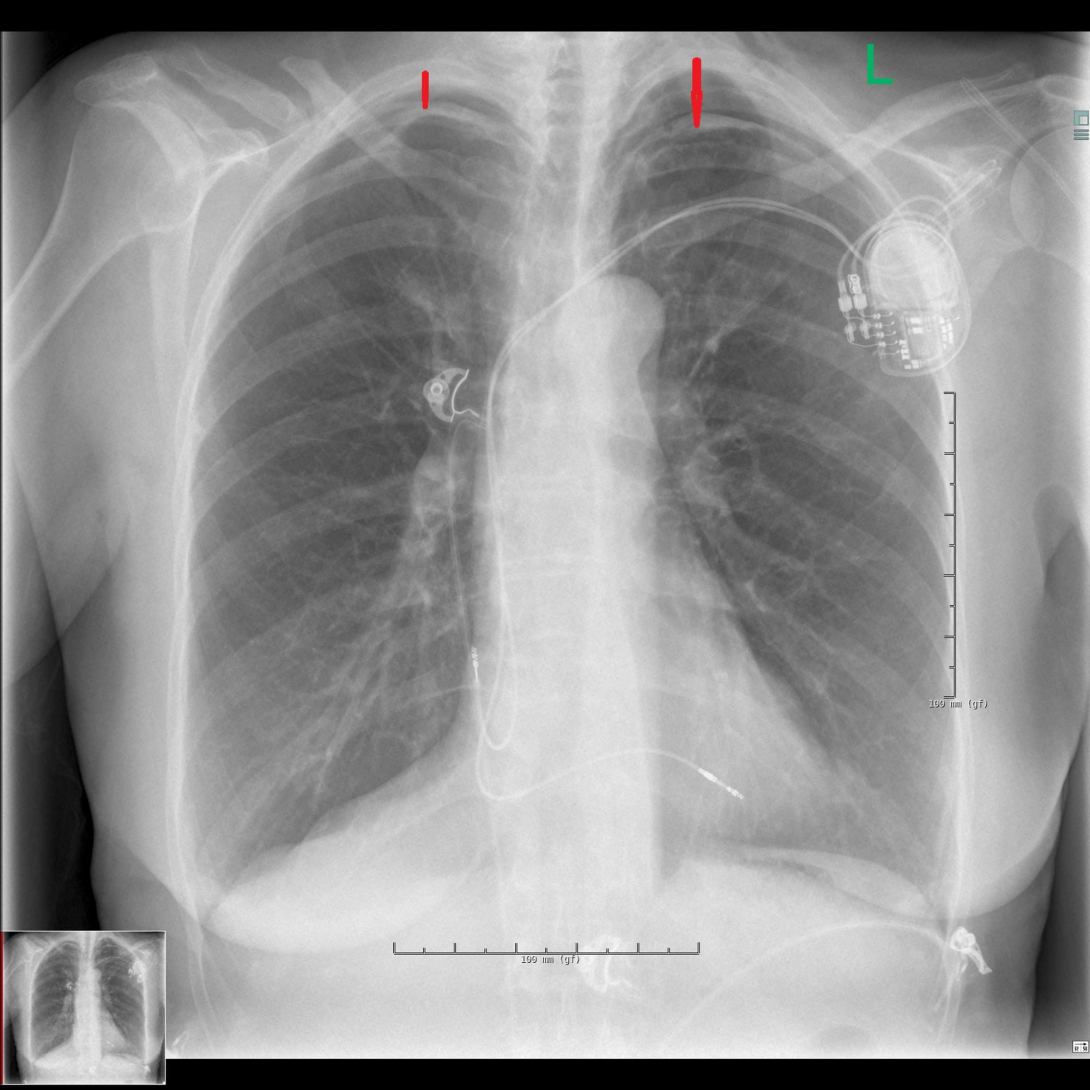
Chest X-ray shows bilateral apical pneumothorax (red arrows) 3 hours after implantation of the pacemaker (day 1).

Later the same day, her chest X-ray showed bilateral apical pneumothorax; it measured 15 mm on the left side, 10 mm on the right side (Figure 2).

She was administered high flow oxygen on a nasal cannula. The patient stayed clinically stable and experienced no breathing problems, no pleuritic chest pain, and no pericardial signs or symptoms.

**Figure 3:**
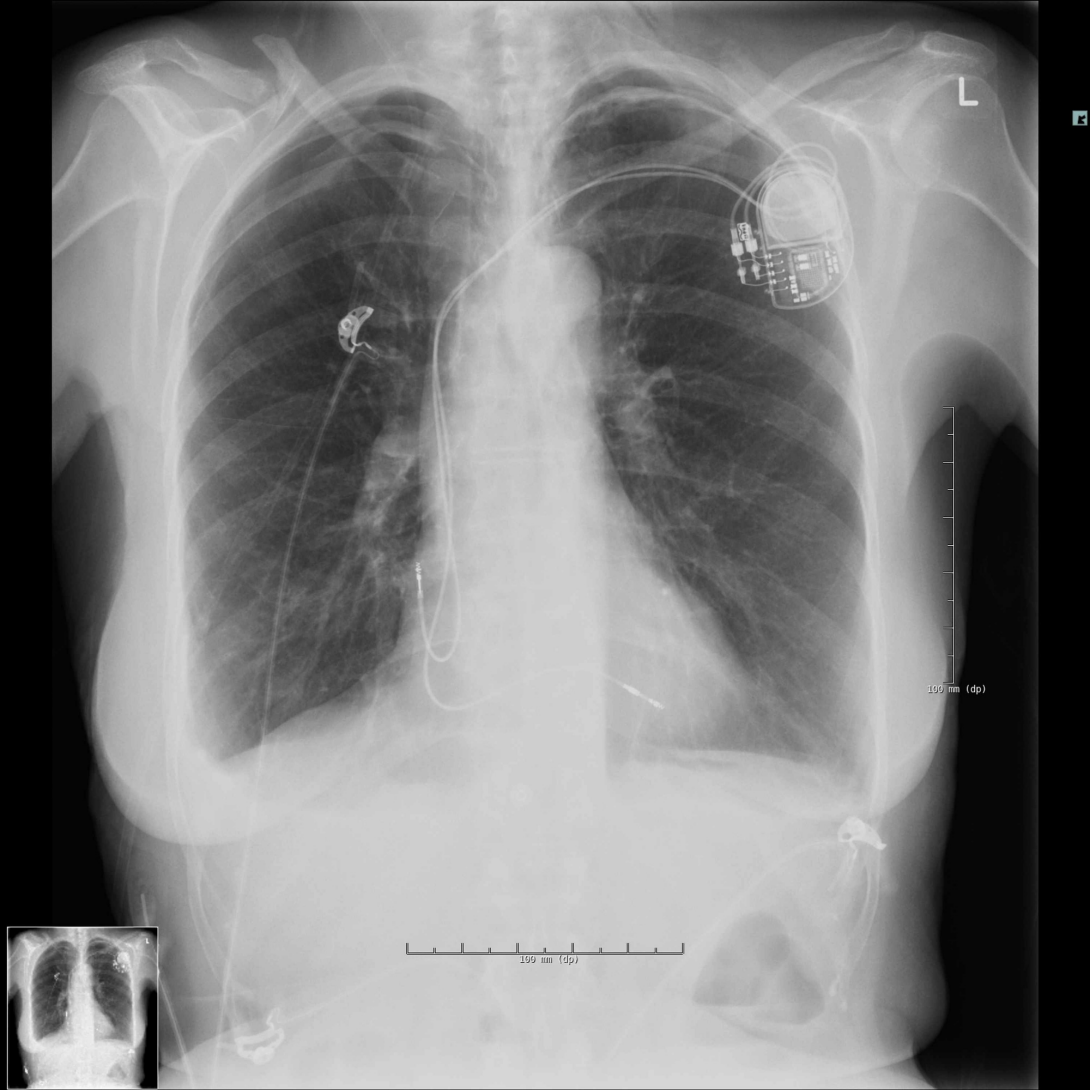
Chest X-ray the day after implantation of the pacemaker (day 2).

The following day, the left pneumothorax had diminished to 7 mm, the right pneumothorax was unchanged, but there was a small effusion in the right costophrenic angle (Figure 3).

**Figure 4:**
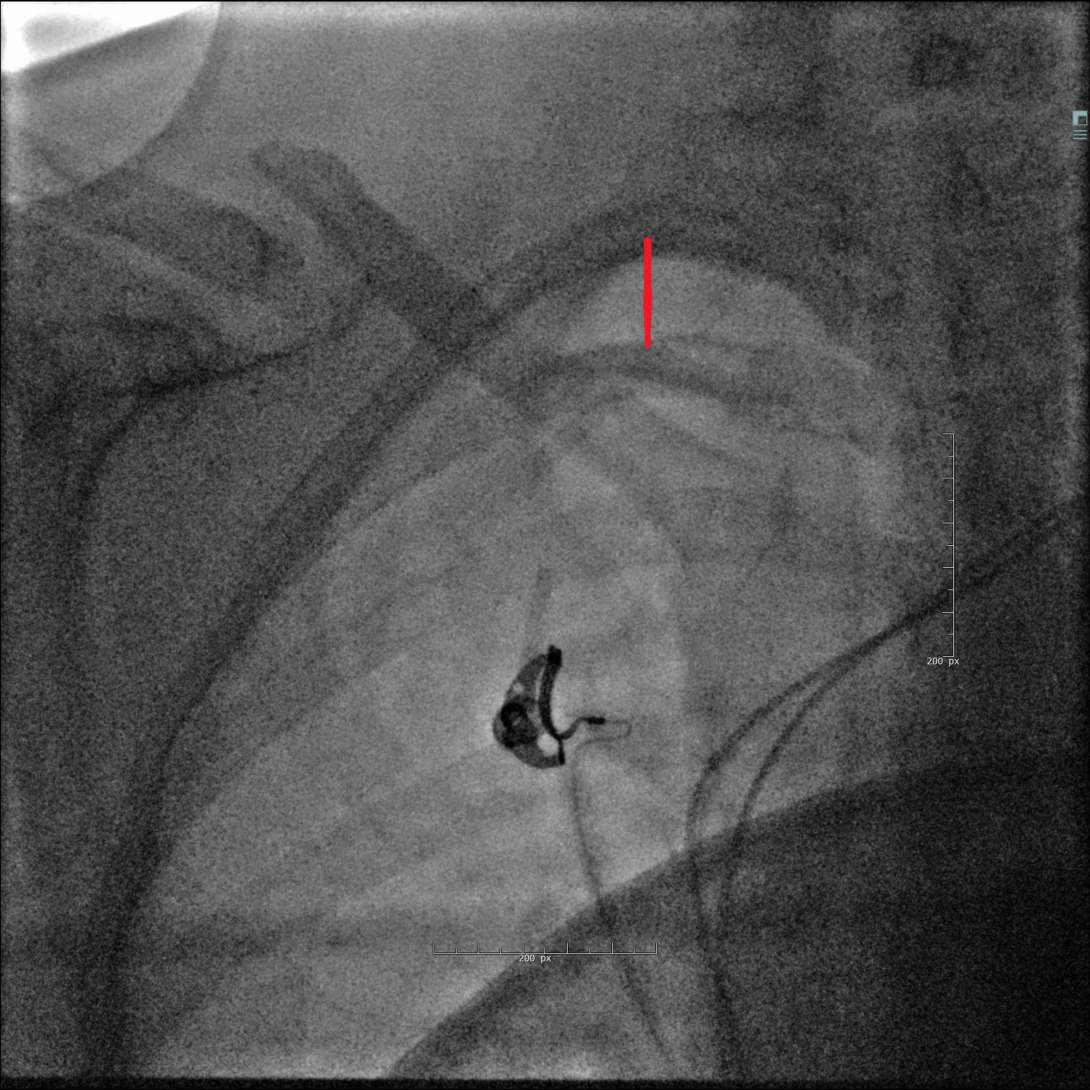
Fluoroscopy shows the right apical pneumothorax on day 2 (red arrow).

**Figure 5:**
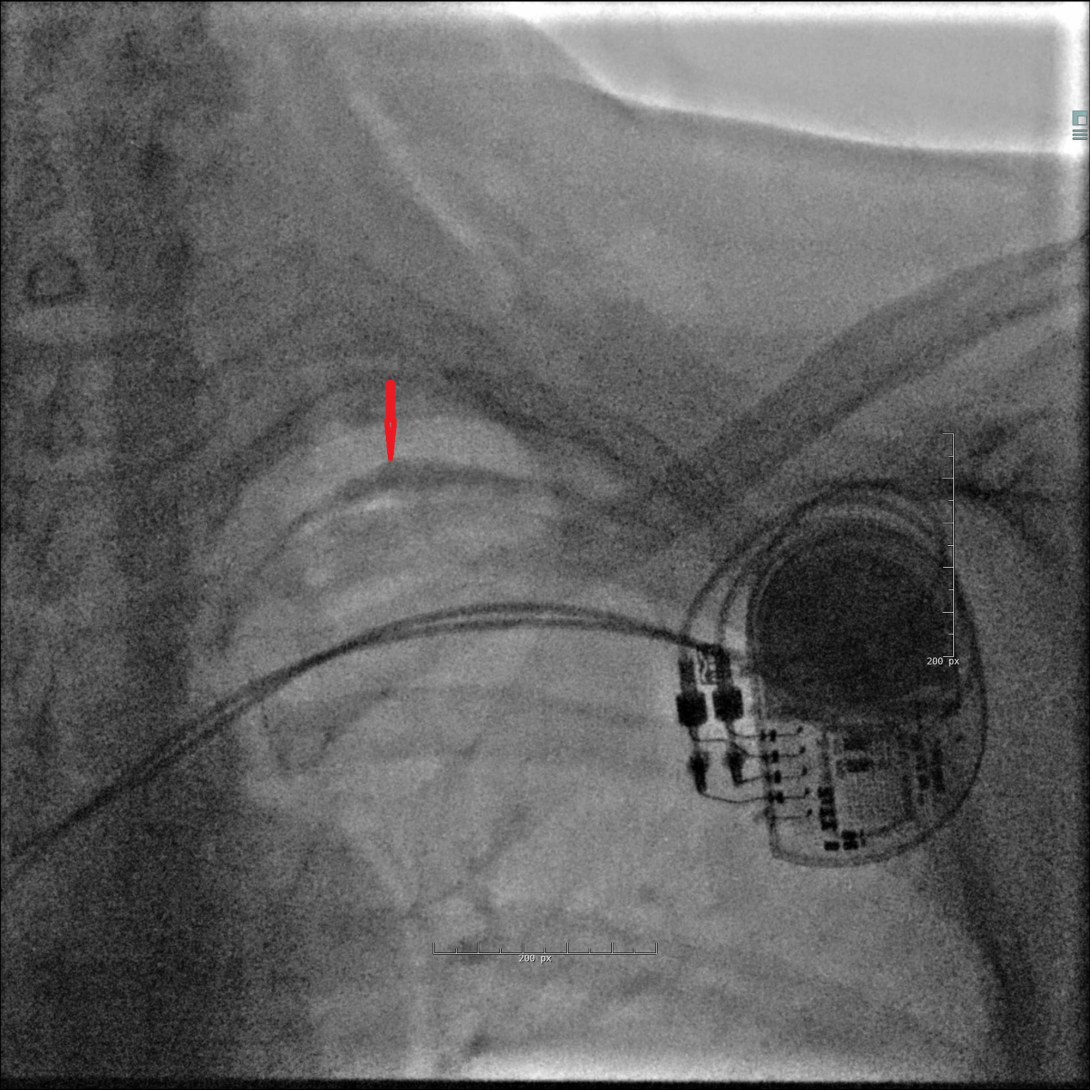
Fluoroscopy shows the left apical pneumothorax on day 2 (red arrow).

**Figure 6:**
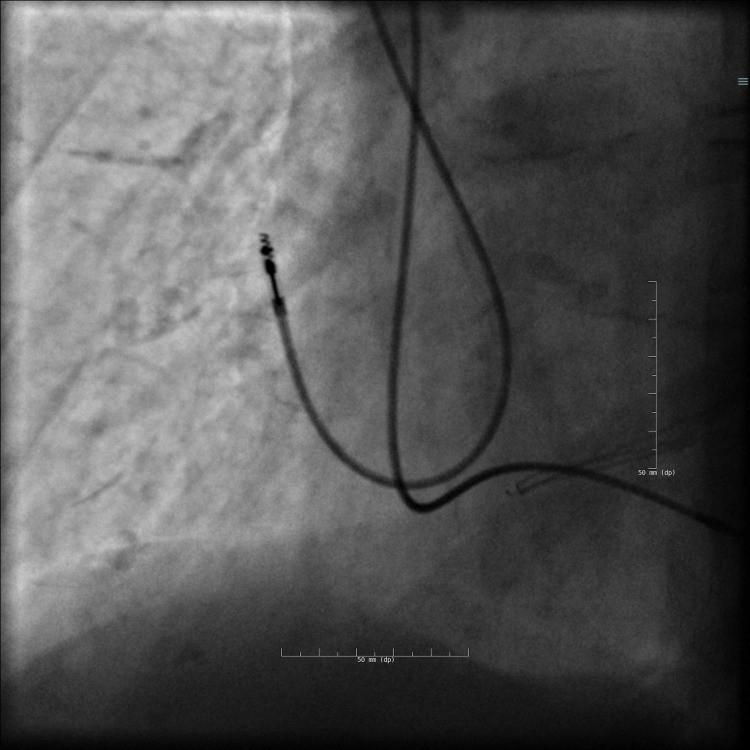
Fluoroscopy on day 2 shows the atrial electrode on the free wall of the atrium.

This was confirmed by fluoroscopy (Figures 4-5). The atrial electrode seemed to respect the atrial wall (Figure 6).

Repeated chest X-ray, fluoroscopy, and echocardiography did not show perforation of the right atrium, and there was no pericardial effusion, no ECG changes, and the pacemaker values remained fine with nonconclusive falls in sense and impedance. The atrial lead parameters at implantation and the following day were: capture 0.8/1 V (duration 0.4 ms); sense 4/2.4 mV, and impedance 400/342 Ohms.

**Figure 7:**
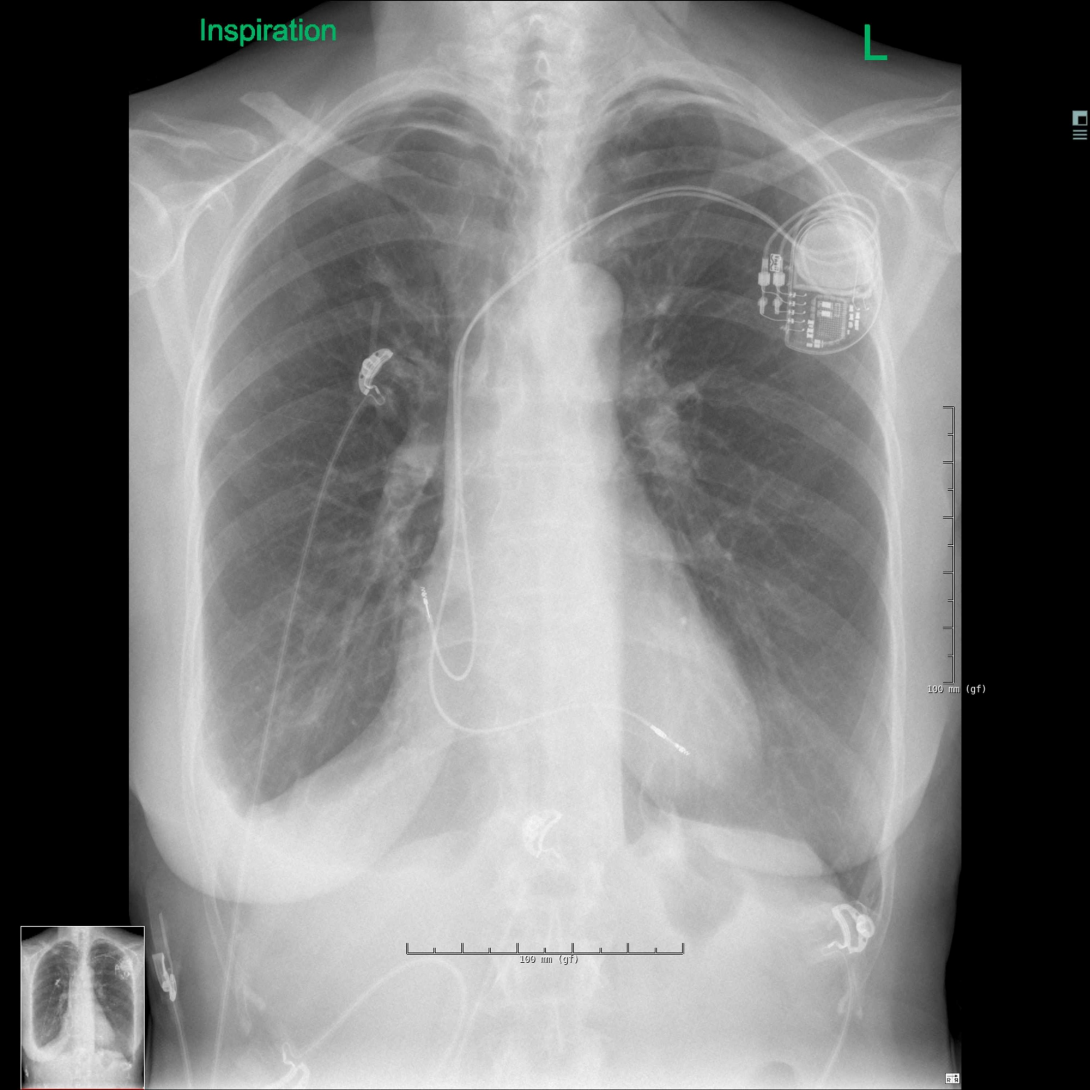
Chest X-ray during inspiration on day 3.

**Figure 8:**
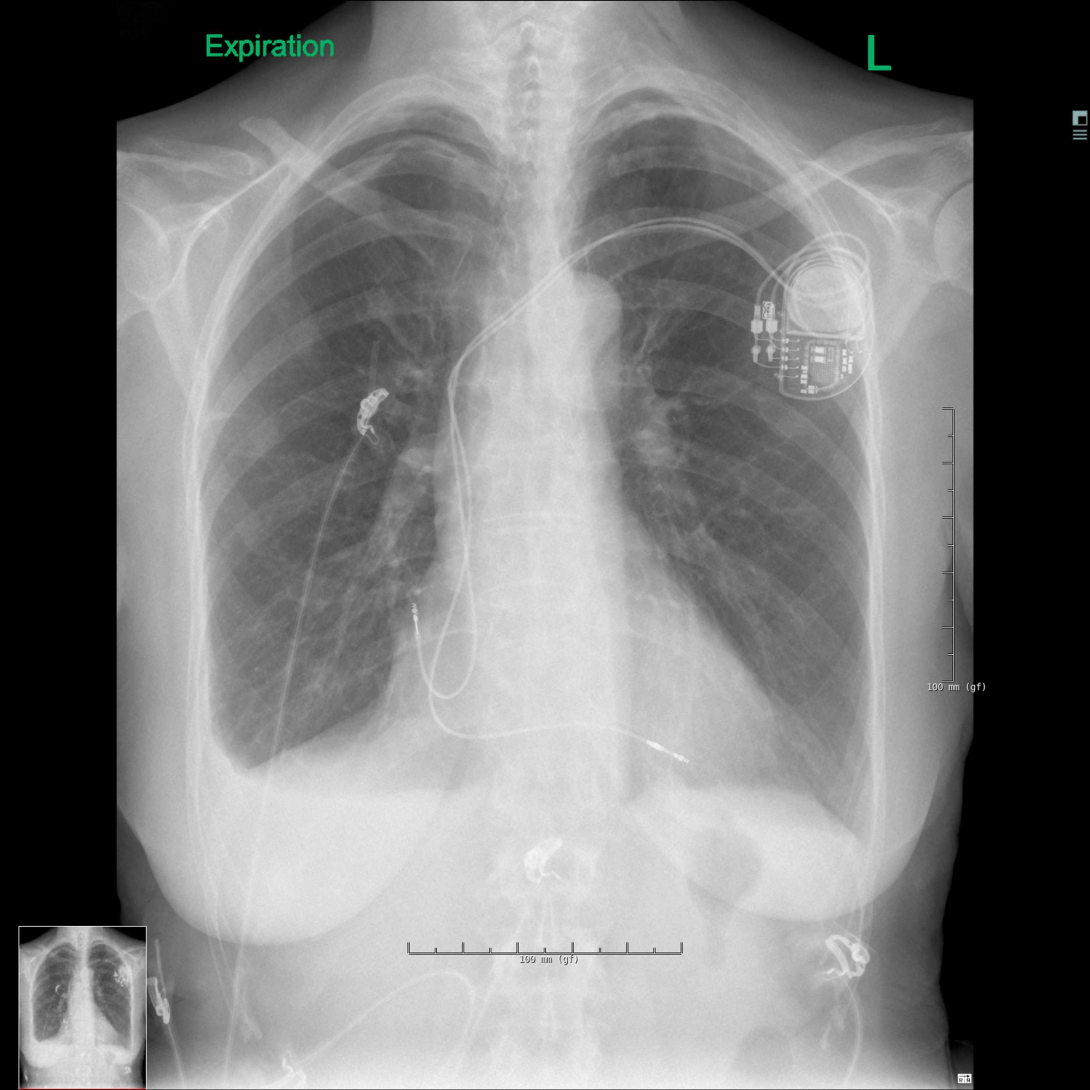
Chest X-ray during expiration on day 3.

**Figure 9:**
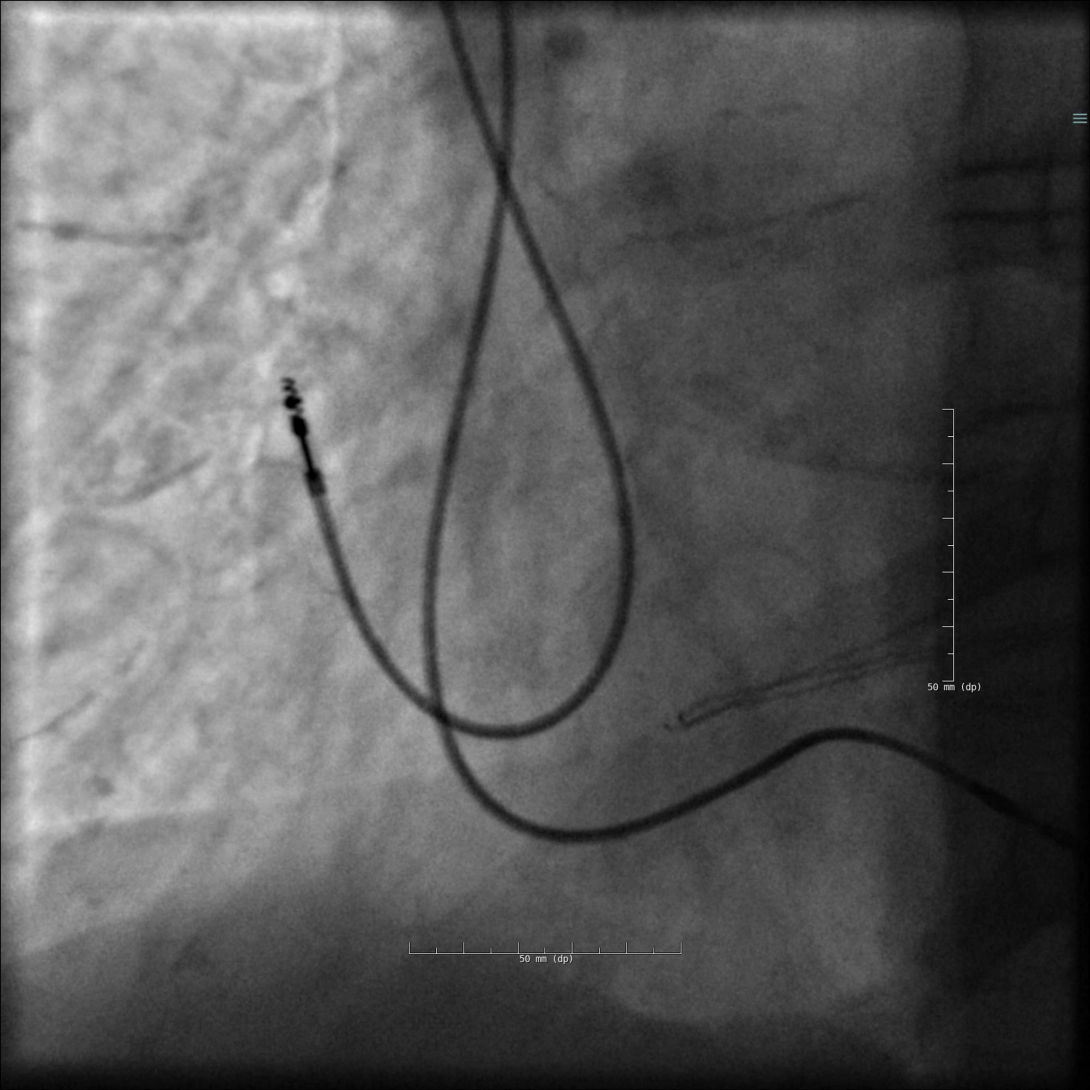
Fluoroscopy on day 3 indicates a stable position of the electrode, which seems to respect the atrial wall.

The following day the left pneumothorax had diminished to 5 mm, but the changes on the right side were the same (Figures 7-8). This led to the decision to replace the atrial electrode, even though the position was unchanged (Figure 9).

The atrial electrode was repositioned; the tip was moved to the right atrial appendage without complications. Echocardiography remained normal.

**Figure 10:**
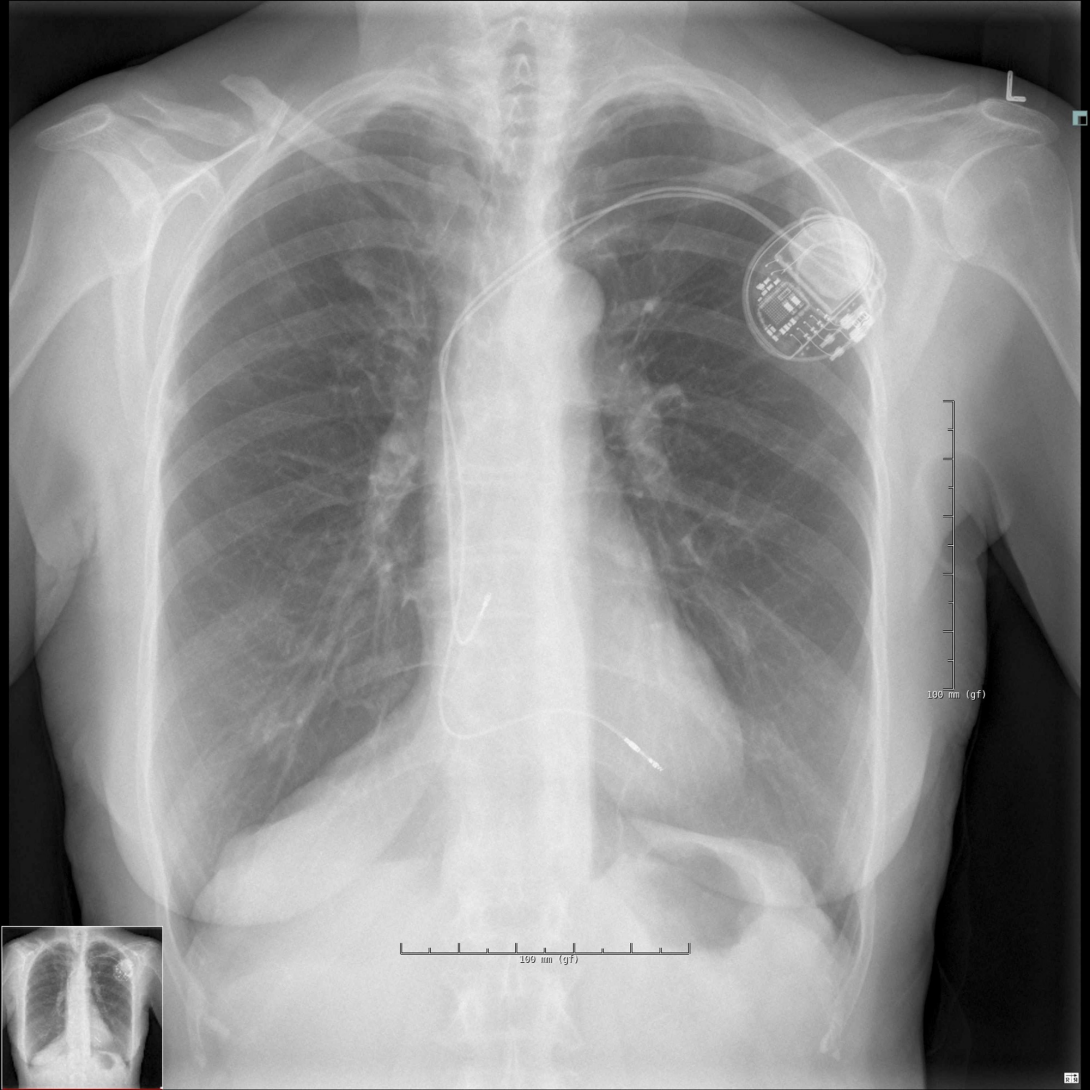
Chest X-ray has normalized 13 days after implantation of the pacemaker.

Two weeks later, the chest X-ray had normalized (Figure 10). The pacemaker was functioning well, and the patient felt fine under the circumstances.

## Discussion

The present case describes an elderly woman with emphysema and a low BMI, who had the pacemaker leads introduced via the subclavian vein, and the atrial electrode positioned on the lateral wall, complicated by simultaneous ipsilateral pneumothorax related to venous access and contralateral pneumothorax due to atrial electrode perforation. Women have a 30% higher risk than men of encountering any complication from pacemaker therapy, mainly due to a 50% higher risk of pneumothorax (2.2%) and cardiac perforation (1.1%) [2]. Shared risk factors for pneumothorax and cardiac perforation besides female gender are BMI<20, age>80 years, chronic obstructive pulmonary disease (COPD), bullous emphysema, corticosteroid treatment, anticoagulation, platelet therapy, emergency procedure, restless and uncooperative patient, and inexperienced operator [2, 5, 8-12].

Ultrasound-guided puncture of the axillary vein or cephalic vein cut-down is preferable to subclavian vein puncture to avoid pneumothorax [5, 13].

The incidence of ipsilateral pneumothorax, due to pleural needle injury when searching for the subclavian vein, averages 2% [5, 10]. The risk of pneumothorax increases with decreasing BMI, from 0.8% in overweight patients to 5.5% in underweight patients. In addition, it is increased in patients with congenital anomalies such as persistent vena cava superior; previous procedures, surgery, trauma, or radiotherapy in the relevant area; deformity of the clavicle; old fractures; chest abnormality; sternotomy; difficult procedure; large catheter size; more than two attempts; long procedure duration; introduction of more than one electrode; system upgrade; and lead revision [1-2, 4-5,10].

In the presence of risk factors, it is recommended to perform venography and use ultrasound guidance or fluoroscopy when searching for the subclavian vein. In patients with a unilateral pulmonary disorder, it is recommended to puncture the subclavian vein on the same side of the affected lung, if possible, as the consequences of potential complications will be less severe. Infraclavicular puncture of the subclavian vein is best performed with the patient in the Trendelenburg position, with a pillow lengthwise along the spine from the scapula to the iliac crest, the head turned away, the neck extended, the chin raised, the shoulder depressed by the left hand, and the left index finger palpating the subclavian artery. This creates a transthoracic arch and allows the insertion of the needle with the syringe parallel to the chest wall thereby minimizing the risk of pneumothorax. The surgeon should aim for the vein component overlying the first rib [1, 10]. The pacemaker-implanting cardiologist should generally stick to the rule never to search for both subclavian veins in sequence, the intention being that when venous access is not possible to obtain, including having thoroughly searched for the subclavian vein on one side, a shift to the opposite side should wait until pneumothorax can be ruled out.

In some cases, pneumothorax may be asymptomatic and in others, it may cause pleuritic pain, dyspnea, tachypnea, and tachycardia [5]. More severe symptoms, like hypoxia (oxygen saturation <90%), hypotension, distended neck veins, displaced trachea, and decreased or absent breath sounds, strongly indicate tension pneumothorax and can be life-threatening [1, 4]. Every time a patient exhibits the classic signs and symptoms associated with pneumothorax within hours of a risky procedure, it should be presumed that the patient experiences a pneumothorax until proven otherwise.

Fluoroscopy, chest X-ray, and ultrasound can sometimes suggest the diagnosis rapidly. CT is the most sensitive approach but results in delayed treatment. The treatment takes precedence over imaging [4,10].

High content of oxygen speeds up the absorption of air from the pleural space and high flow nasal oxygen (10 L) should be provided. A small pneumothorax (<3 cm) in a stable patient should be observed without drainage because it may resolve spontaneously. If the patient is unstable, it is important to reduce excess air in the pleural space, and drainage should be performed [10]. Chest tubes should be inserted in either the second or the third intercostal space of the midclavicular line; alternatively, anterior to the mid-axillary line of the fourth or fifth intercostal space [4]. The incidence of pneumothorax requiring a chest tube was 0.66% in a population-based Danish cohort study [2].

As the patient had no prior history of hospitalization or prior surgery, it is most unlikely that this case of bilateral pneumothorax should be due to interpleural communication (Buffalo chest), which is a rare condition that can be congenital, traumatic, or iatrogenic following cardiothoracic or laparoscopic surgery [10].

The most plausible explanation of the right pneumothorax was considered to be perforation of the right atrium and pericardium and lung injury. It is likely that the perforation was small, plugged spontaneously, and happened while searching for a good atrial position, prior to finally fixing the tip of the atrial lead on the lateral wall. This could explain why lead perforation of the atrial wall could not be identified. With this in mind, and based on the literature describing conservative management of similar cases, the patient was kept under close observation for 24 hours. The opinion changed due to the persistence of the right pneumothorax on the third day, even though it did not necessarily imply causality by the atrial lead being persistently perforated. It was decided to give the patient the benefit of the doubt and replace the electrode. This is the commonly recommended strategy.

If the defect caused by perforation of the cardiac wall is small and plugged immediately due to cardiomyocyte contraction, then a pericardial effusion is avoided and the pneumothorax can be stabilized. A larger cardiac perforation can lead to pericarditis, pericardial effusion, tamponade, hemodynamic instability [14] and even death; pleuritis, chest pain, dyspnea, hypotension, and syncope [12, 15-18], abnormal pacemaker function (primarily a high threshold or loss of capture) [9, 11-12, 14-17, 19] and extracardiac pacing (diaphragmatic, pectoralis or intercostal muscle stimulation [14-15, 19] and inappropriate shock [17]. Pneumopericardium and pneumothorax and pleural effusion are very rare complications [3, 6-9, 15, 17]. The patient may be asymptomatic and the pacemaker interrogation may be normal, despite lead perforation [19].

Perforation, with the lead or the helix outside the cardiac silhouette, can be diagnosed by means of fluoroscopy, chest radiography, and echocardiography [14, 19], but these methods are not reliable for evaluating less severe lead perforation [8-9, 17, 20]. The diagnostic gold standard is ECG-gated high-resolution computed tomography (HRCT) [9], which has optimal demarcation of the interface between the myocardium, blood, and fat [12, 15, 17-20], although star artifacts from the pacemaker wire sometimes make it difficult to precisely identify the lead tip [8-9, 17, 19-20], and there is a risk of over-diagnosing perforation [16]. Apart from aiding in making the diagnosis HRCT also helps in planning lead retrieval as it gives a good assessment of the orientation of vital structures around the displaced lead [9, 16].

The atrial electrode is more prone than the ventricular electrode to perforate the myocardium as the atrial wall is thin, about 2 mm thick, and maybe atrial free wall position carries a higher risk than the appendage. Likewise, ventricular perforation mostly occurs in the relatively thin-walled apex or the free wall of the right ventricle. A septal position is preferred [14].

To minimize the risk of perforation, the surgeon should abstain from excessive advancement of the helix during lead fixation, excessive pressure on the lead, excessive loop, and forced dislodgement of the lead with an extended screw for instance due to dislocation of the atrial lead during the insertion of a ventricular lead, which should therefore be positioned first. Additional risk factors for cardiac perforation are variations in anatomy, persisting left superior vena cava, thin-wall due to muscular dystrophy, dilated and ischemic cardiomyopathy, difficult procedure, concomitant use of a temporary transvenous pacemaker and repeated repositioning of the lead. Also, lead factors (lead thickness and the design and stiffness of the helix) may be of importance. Leadless pacemakers seem to be associated with a slightly higher perforation risk. Implantable cardioverter-defibrillator (ICD) leads have a larger diameter and a higher rate of perforation, whereas cardiac resynchronization therapy defibrillator (CRTD)-implantation is associated with the highest risk of perforation [3, 6-8,11-12,14-17].

Protective factors against perforation are hypertrophy, elevated high ventricular systolic pressure >35 mmHg, previous cardiac surgery, and BMI >30 [12,15]. 

Clinically relevant and symptomatic perforation of the cardiac wall occurs mostly within one month after implantation and in 0.3%-1.2% of all pacemaker procedures [6, 11-12, 15]. Surprisingly, pericardial effusion was seen in 10% when newly implanted patients underwent a systematic echocardiographic evaluation, but it resolved spontaneously and rarely required therapy although most cases were probably caused by perforation of an implanted lead [11]. Correspondingly, late perforation was seen in 15% of CT-scanned asymptomatic pacemaker patients and in 27% of postmortem examinations. These findings raise the potential of underdiagnosing lead perforation [6-7, 11-12, 17-18]. 

Appropriate management of asymptomatic lead perforation is a debated issue [16-19]. When there is a regression of the pneumothorax, no pericardial effusion, no clinical symptoms, and stable and acceptable lead parameters it may be considered to closely observe the patient with a conservative approach and to retain the electrode in situ [6-7], especially in the old and frail patient with an increased risk associated with lead extraction [15-16]; even in cases with helix perforation, awaiting fibrous tissue thickening and/or wrapping around the helix [17]. The long-term safety of this strategy is unknown [12, 18-19]. However, there is a risk of progressive perforation of the pacing lead and serious damage to the surrounding tissue, structures, and organs [16,19]. For this reason, it is generally recommended to extract or to reposition the lead [12, 16, 18-20]. In acute and subacute perforation transvenous lead extraction is commonly possible without any difficulties, and in most cases, the lead can be safely extracted under fluoroscopic guidance with hemodynamic and echocardiographic monitoring; placement of a prophylactic pericardial drain catheter or preparedness to perform emergency pericardiocentesis; plus backup from a cardiosurgical team ready to perform thoracotomy with surgical extraction and lead removal [8, 12, 15-17, 19]. Because of the increased risk of infections and constrictive pericarditis close follow-up is recommended [19].

## Conclusions

The first reported case about simultaneous bilateral pneumothorax complicating implantation of a dual-chamber pacemaker; pleural injury caused ipsilateral by needle puncture when searching for the subclavian vein, and contralateral by perforation of the atrial electrode; in a high-risk old, frail, underweight, restless female with pulmonary emphysema.

To prevent pneumothorax, puncture of the subclavian vein should be guided by venography and fluoroscopy; extrathoracic venous access should be preferred (ultrasound-guided puncture of the axillary vein or cut down of the cephalic vein); and the electrode should preferably not be positioned in the free wall.
